# Radiographic Sagittal Alignment and Kinetic Chain Alterations in Geriatric Patients With Scoliosis: A Case Series

**DOI:** 10.7759/cureus.105827

**Published:** 2026-03-25

**Authors:** John P Whelan, Justin M Dick

**Affiliations:** 1 Physical Medicine and Rehabilitation, Clear Life Scoliosis and Chiropractic Center, Charlotte, USA

**Keywords:** adolescent idiopathic scoliosis (ais), adult idiopathic scoliosis, complete kinetic chain, geriatric physical therapy, non-surgical rehabilitation, spinal degeneration

## Abstract

Adolescent idiopathic scoliosis (AIS) is a three-dimensional spinal deformity that originates in adolescence but carries lifelong biomechanical consequences. In geriatric patients, chronic pain associated with a history of AIS is frequently attributed to the scoliotic curve itself. This case series proposes an alternative framework. Pain in this population may originate from long-term kinetic chain dysfunction and accelerated degeneration driven by decades of compensatory biomechanical loading.

Two geriatric subjects with a history of AIS and chronic pain presented to a private chiropractic clinic. Both underwent a multimodal non-surgical spinal rehabilitation program based on the CLEAR Institute protocol. Pre- and post-treatment radiographic measurements and self-reported outcomes were assessed. Both patients demonstrated clinically meaningful reductions in pain and measurable improvements in sagittal spinal alignment following treatment.

These cases suggest that in geriatric patients with AIS, chronic deficits may be more closely related to kinetic chain dysfunction and compensatory degeneration than to the primary scoliotic curve. A non-surgical, multimodal rehabilitation approach targeting global spinal alignment can yield significant clinical improvement. Larger prospective studies are needed to evaluate this hypothesis further.

## Introduction

Adolescent idiopathic scoliosis (AIS) is a structural, three-dimensional spinal deformity defined by a lateral curvature of 10 degrees or greater accompanied by vertebral rotation and trunk asymmetry [1]. It typically presents between the ages of 10 and 17 and affects approximately 2 to 3 percent of adolescents, with a marked predominance in females [2,3]. Curve progression most commonly coincides with periods of rapid pubertal growth. Left untreated, AIS can lead to pain, progressive deformity, and psychological distress related to body image and disability [3].

The long-term natural history of AIS extends well beyond skeletal maturity. Spinal curves, particularly those exceeding 50 degrees, have been shown to continue progressing into adulthood [4]. The prevalence of scoliosis in the general population increases sharply with age. Population studies report rates as high as 68 percent in individuals between 60 and 90 years old, a figure that includes both de novo degenerative scoliosis and the progression of pre-existing AIS [5]. This convergence of adolescent deformity and age-related degeneration creates a clinically complex presentation in older adults. The primary source of pain in these patients is often difficult to determine.

Scoliosis should also be understood within the context of the kinetic chain. The cervical spine, thoracic spine, lumbar spine, pelvis, and lower extremities function as an integrated biomechanical unit. A structural deformity in one spinal region does not remain regionally confined. It obligates compensatory adaptations throughout the entire chain to preserve sagittal balance, horizontal gaze, and ambulatory function. These compensations may involve altered cervical curvature, thoracolumbar junctional remodeling, pelvic obliquity, asymmetric lower-extremity loading, and gait deviations. Such strategies will become maladaptive over time. This results in chronic asymmetric loading across discs, facet joints, ligaments, and myofascial tissues. This persistent mechanical inefficiency may accelerate degenerative change, promote segmental instability, and contribute to progressive pain and dysfunction in areas both adjacent to and distant from the primary scoliotic deformity. Published literature demonstrates that patients with adolescent idiopathic scoliosis frequently exhibit abnormal cervical mechanics, including hypolordosis, instability, and cervical buckling patterns, suggesting that the biomechanical impact of scoliosis extends well beyond the primary coronal curve [6]. As these compensatory patterns accumulate over decades, they may progressively reduce functional reserve and impair the body’s ability to tolerate normal mechanical demands. This decline can contribute to diminished tolerance for standing, walking, lifting, and other activities of daily living. Additional evidence has documented that restoration of sagittal spinal alignment in patients with complex spinal histories, including prior surgery and pre-existing scoliosis, is associated with meaningful reductions in pain and disability [7,8].

This case series aims to describe the clinical presentation, radiographic findings, and treatment outcomes of two geriatric female patients with a history of AIS who presented with chronic functional deficits. We propose that deficits were not only a direct consequence of the scoliotic curve but rather the result of long-term kinetic chain dysfunction. Both patients presented consecutively for scoliosis-specific care using the CLEAR Institute treatment protocols and monitored with a subjective functional rating index and objective radiographs [1,9-13].

## Case presentation

Case 1

A 74-year-old female presented with a known history of AIS and chronic lower back pain with intermittent radiation into her right lower extremity. She rated her pain as a 6 out of 10 on a numeric pain scale. The pain was a constant dull ache that worsened with prolonged standing or walking. The patient's gait was severely disrupted. She used a walking cane to assist in ambulation and reported a history of falls and a subjective feeling of instability with ambulation. No prior care was tried for her scoliosis or gait abnormalities. Surgical intervention was recommended. Her primary goals were to manage her pain and improve her balance.

Physical examination revealed a left lumbar prominence and mild trunk shift to the left. Paraspinal musculature was hypertonic in the left lumbar region. Bilateral piriformis syndrome was noted upon palpation, which increased pain. Bilateral posterior superior iliac spine (PSIS) palpation induced pain. Postural assessment demonstrated a forward head posture. Anterior hip rotation was noted on the posture evaluation. Orthopedic testing was negative for significant radiculopathy on examination. She reported intermittent lower extremity radicular symptoms with walking (Table 1).

**Table 1:** Objective clinical and functional measurements at baseline and visit 18 of 20 Objective clinical and functional outcomes were assessed at baseline and again at the eighteenth visit. Functional status was evaluated using the functional rating index (FRI), a validated patient-reported outcome measure that combines elements of the Oswestry Low Back Disability Questionnaire and the Neck Disability Index into a single instrument designed to assess spinal-related disability while reducing administrative burden. The FRI is scored on a scale from 0 to 40, with lower scores indicating minimal functional impairment and higher scores reflecting greater disability. Previous investigations have demonstrated that the FRI exhibits strong reliability, validity, and responsiveness, supporting its use in both clinical practice and research settings.

Measure	Case 1 Baseline (June 7, 2023)	Case 1 Visit 18 (Sept 7, 2023)	Case 2 Baseline (Feb 1, 2024)	Case 2 Visit 18 (Apr 4, 2024)
Functional Rating Index	19	12	12	7
Height (cm)	166.5	166.5	166.5	166.5
Chest expansion (in)	1.5	3	1	1.75
Cervical Flexion Test (in)	1.5	0	1	0.75
Angle of trunk rotation – dorsal (flexion) Right	7°	0°	0°	0°
Angle of trunk rotation – dorsal (flexion) Left	0°	0°	4°	6°
Angle of trunk rotation – dorsal lumbar (flexion) Right	0°	0°	0°	1°
Angle of trunk rotation – dorsal lumbar (flexion) Left	4°	0°	0°	0°
Angle of trunk rotation – lumbar (flexion) Right	7°	9°	10°	0°
Angle of trunk rotation – lumbar (flexion) Left	0°	0°	0°	0°
Angle of trunk rotation – dorsal (prone) Right	10°	0°	0°	0°
Angle of trunk rotation – dorsal (prone) Left	0°	0°	7°	7°
Angle of trunk rotation – dorsal lumbar (prone) Right	10°	0°	0°	0°
Angle of trunk rotation – dorsal lumbar (prone) Left	0°	0°	1°	1°
Angle of trunk rotation – lumbar (prone) Right	15°	9°	21°	21°
Angle of trunk rotation – lumbar (prone) Left	0°	0°	0°	0°
Balance test (seconds to failure) – Left	15	WNL	WNL	WNL
Balance test (seconds to failure) – Right	0	4	WNL	WNL
Stork test (seconds to failure) – Left	0	3	3	10
Stork test (seconds to failure) – Right	0	0	7	7
Modified Cox test (degrees) – Left	60	60	45	70
Modified Cox test (degrees) – Right	60	85	70	80

Intervention

Following an initial evaluation, Case 1 completed a spinal corrective program consisting of 20 in-office sessions (3 sessions per week spanning 7 consecutive weeks). The CLEAR™ Institute’s “Mix-Fix-Set” model of care was utilized. Each session lasted approximately 120 minutes and was supervised by a licensed chiropractor trained in scoliosis biomechanics through the CLEAR™ Institute.

Results

Paraspinal musculature continued to be hypertonic in the left lumbar region. Pain associated with previously documented bilateral piriformis syndrome was reduced. Bilateral PSIS palpation did not induce pain. She reported the ability to ambulate without the use of a cane. She reported that she felt more "stable" on her feet. She reported she could resume activities of daily living but was unable to farm.

**Figure 1:**
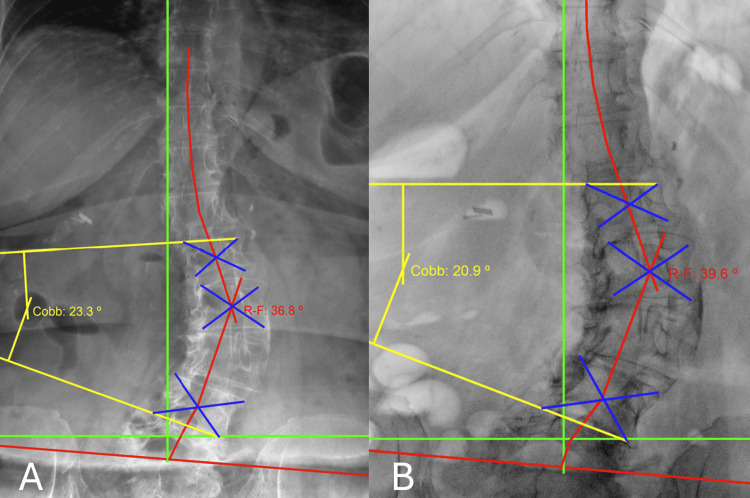
Case 1: Anterior-posterior (AP) radiographs were obtained at 2 intervals: pre-treatment (Panel A), prior to care on visit 18 (Panel B) Panel A represents AP radiographs on 07/10/2023. Panel B represents AP radiographs, on09/07/2023. The yellow lines indicate Cobb angle measurements. The green lines represent the ideal coronal alignment reference. The red lines illustrate the Risser-Ferguson analysis. Serial imaging demonstrates progressive improvement in the magnitude of the Cobb angle.

Radiographic evaluation was performed on July 10, 2023, of weight-bearing radiographs. The anteroposterior view demonstrated moderate left lumbar scoliosis. The Cobb angle measured 23.3 degrees at initial presentation and reduced to 20.9 degrees (Figure 1, Table 2). Quantitative cervical and lumbopelvic spine changes were measured to assess structural outcomes utilizing PostureRay (PostureCo, Inc., Trinity, FL). This is a machine learning-assisted parameter measurement. Radiographic line-drawing methods have demonstrated high inter- and intra-examiner reliability and are comparable to manual measurements across spinal regions. Measurements were compared with normative values and baseline imaging to determine the degree of anatomical correction. All images and analysis were completed by the same physician.

**Table 2:** Case 1: Comparison of anterior-posterior (AP) radiographs X-ray 1 was acquired on 07.10.2023. X-ray 2 was acquired on 09.07.2023. The Risser-Ferguson angle is determined by identifying the most tilted vertebra at the superior and inferior ends of the curve and the vertebra located at the apex of the curvature. Lines are then drawn connecting the centers of these vertebral bodies. The angle formed between these lines represents the magnitude of the spinal curve. The Cobb angle is calculated by drawing lines along the superior endplate of the upper end vertebra and the inferior endplate of the lower end vertebra of the curve. This is followed by constructing perpendicular lines from these endplate lines. The angle formed at the intersection of these perpendiculars represents the Cobb angle.

Metric	Normal Values	X-ray 1 Values	Difference From Normal (X-ray 1)	X-ray 2 Values	Difference From Normal (X-ray 2)	% Change X-ray 1→2
Risser-Ferguson Angle L1-L4 (L2)	0º	36.8º	36.8º	39.6º	39.6º	7.6%
Cobb Angle L1-L4	0º	23.3º	23.3º	20.9º	20.9º	10.3%

**Figure 2:**
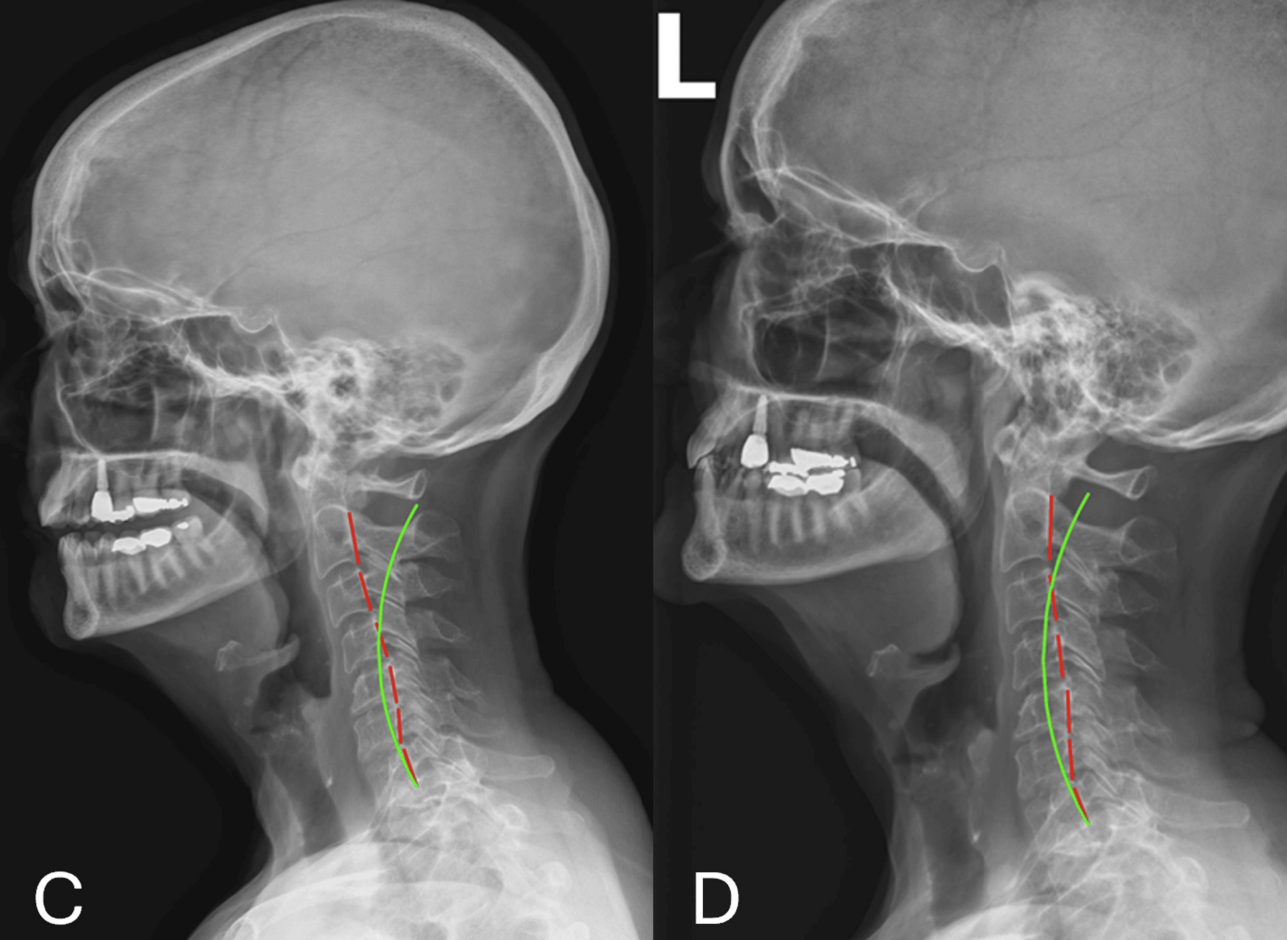
Case 1: Lateral cervical neutral radiographs were obtained at two intervals: pre-treatment (Panel C), prior to care on visit 18 (Panel D) Panel C represents lateral cervical neutral on 07/10/2023. Panel D represents lateral cervical neutral on 09/07/2023. The green reference line illustrates the expected posterior vertebral body alignment corresponding to an established normal course of the posterior longitudinal ligament. The red reference line represents the subject's measured alignment using George's line. This indicates a deviation from ideal posterior vertebral body positioning.

Sagittal analysis of the lateral cervical projection revealed a cervical kyphosis. The absolute rotation angle (ARA) of C2-C7 measured -9.2 degrees, a significant deviation from the normative value of -42.0 degrees. Forward head posture measured 25.9 mm of anterior translation (Figure 2, Table 3). These findings demonstrate a complex, multi-regional spinal deformity with significant sagittal and coronal imbalances, consistent with loss of cervical lordosis and age-related degenerative progression.

**Table 3:** Case 1: Comparison of lateral cervical neutral radiographs X-ray 1 was acquired on 07.10.2023. X-ray 2 was acquired on 09.07.2023. The absolute rotation angle (ARA) is a radiographic measurement used to quantify the overall curvature of a spinal region on lateral radiographs. It is calculated by measuring the angle formed between posterior tangent lines drawn along the vertebral bodies at the superior and inferior limits of the spinal segment being evaluated, providing an estimate of the total sagittal curvature of that region.

RRA Segment	Normal Values	X-ray 1 Values	Versus Normal (X-ray 1)	X-ray 2 Values	Versus Normal (X-ray 2)	% Change X-ray 1→2
ARA C2-C7	-42.0º	-9.2º	78.10%	-22.9º	45.50%	148.90%
Translation C2-C7	0.0 mm	25.9 mm	25.9 mm	11.9 mm	11.9 mm	54.10%

Case 2

A 60-year-old female presented with a long-standing history of idiopathic scoliosis. The patient reported chronic debilitating lower back pain with ambulation only. She rated her pain as 0 out of 10 on a numeric pain scale statistically. Her abnormal gait was constant and significantly limited her ability to perform daily activities, as this induced pain. Surgical intervention was recommended. No prior care was tried for her scoliosis or gait abnormalities.

Physical examination revealed significant trunk asymmetry with a right thoracic prominence and an elevated right shoulder. Paraspinal musculature was hypertonic bilaterally, with greater tension on the right (Table 1). Bilateral piriformis syndrome was noted with increased pain upon palpation. Bilateral PSIS palpation induced pain. Postural assessment demonstrated a forward head posture. Anterior hip rotation was noted on the posture evaluation. Orthopedic and neurological testing was otherwise unremarkable. The patient's gait was severely disrupted.

Intervention

The patient completed a two-month course (three sessions per week spanning seven consecutive weeks) of scoliosis-specific chiropractic care following the CLEAR Institute protocol. This multimodal program incorporated spinal adjustments, scoliosis-specific rehabilitative exercises, and spinal traction. The CLEAR protocol is a structured, evidence-informed approach to non-surgical scoliosis management that integrates mechanical traction, proprioceptive neuromuscular re-education, and corrective exercise to address the three-dimensional nature of scoliotic deformity.

Results

Paraspinal musculature continued to be hypertonic in the left lumbar region. Previously noted bilateral piriformis syndrome demonstrated reduced pain. Bilateral PSIS palpation did not induce pain. Clinically, she reported the ability to walk without pain for longer distances. She also reported a marked improvement in her ability to perform daily activities while standing.

**Figure 3:**
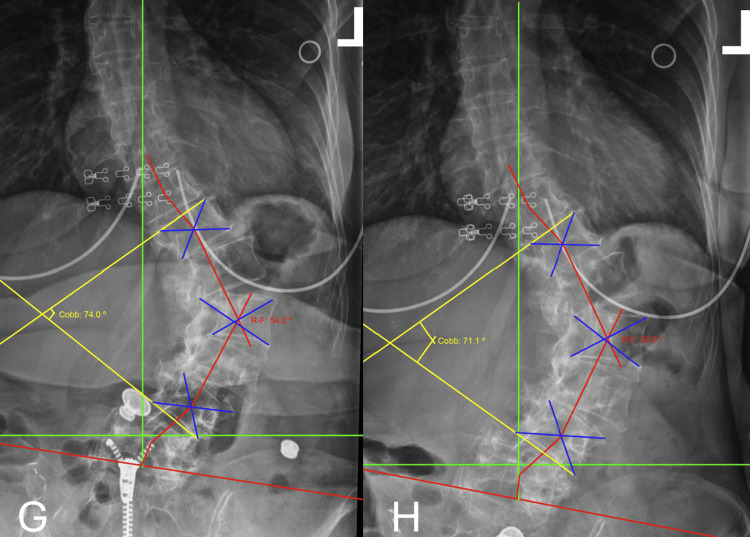
Case 2: Anterior-posterior (AP) radiographs were obtained at 2 intervals: pre-treatment (Panel G) and prior to care on visit 18 (Panel H) Panel G represents AP radiographs on 02/05/2024. Panel H represents AP radiographs on 04/04/2024. The yellow lines indicate Cobb angle measurements. The green lines represent the ideal coronal alignment reference. The red lines illustrate the Risser-Ferguson analysis. Serial imaging demonstrates progressive improvement in the magnitude of the Cobb angle

Follow-up radiographs were obtained on April 4, 2024. The thoracolumbar Cobb angle reduced from 74.0 degrees to 71.1 degrees (Figure 3, Table 4). 

**Table 4:** Case 2: Comparison of anterior-posterior (AP) radiographs X-ray 1 was acquired on 02/05/2024. X-ray 2 was acquired on 04/04/2024. The Risser-Ferguson angle is determined by identifying the most tilted vertebrae at the superior and inferior ends of the curve and the vertebra located at the apex of the curvature. Lines are then drawn connecting the centers of these vertebral bodies. The angle formed between these lines represents the magnitude of the spinal curve. The Cobb angle is calculated by drawing lines along the superior endplate of the upper end vertebra and the inferior endplate of the lower end vertebra of the curve. This is followed by constructing perpendicular lines from these endplate lines. The angle formed at the intersection of these perpendiculars represents the Cobb angle.

Metric	Normal Values	X-ray 1 Values	Difference From Normal (X-ray 1)	X-ray 2 Values	Difference From Normal (X-ray 2)	% Change X-ray 1→2
Risser-Ferguson Angle T12-L4 (L2)	0º	54.2º	54.2º	50.5º	50.5º	6.8%
Cobb Angle T12-L4	0º	74.0º	74.0º	71.1º	71.1º	3.9%

**Figure 4:**
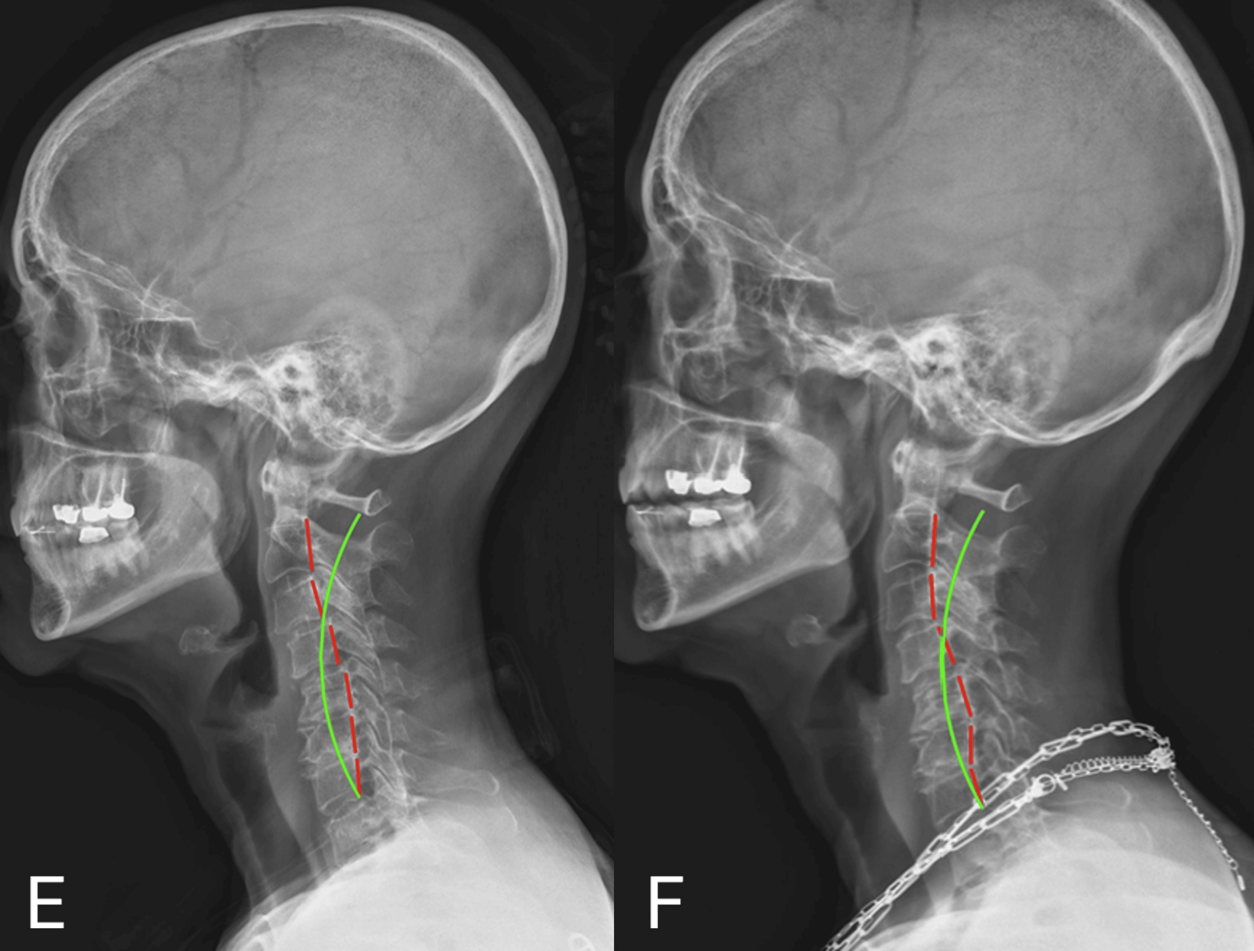
Case 2: Lateral cervical neutral radiographs were obtained at 2 intervals: pre-treatment (Panel E), prior to care on visit 18 (Panel F) Panel E represents lateral cervical neutral on 02/05/2024. Panel F represents lateral cervical neutral on 04/04/2024. The green reference line illustrates the expected posterior vertebral body alignment corresponding to an established normal course of the posterior longitudinal ligament. The red reference line represents the subject's measured alignment using George's line. This indicates a deviation from ideal posterior vertebral body positioning.

The cervical ARA improved from 2.1 degrees to -20.4 degrees, representing a normalization toward the expected lordotic curvature. Forward head posture decreased from 19.7 mm to 15.5 mm (Figure 4, Table 5).

**Table 5:** Case 2: Comparison of lateral cervical neutral radiographs X-ray 1 was acquired on 02.05.2024. X-ray 2 was acquired on 04.04.2024. The absolute rotation angle (ARA) is a radiographic measurement used to quantify the overall curvature of a spinal region on lateral radiographs. It is calculated by measuring the angle formed between posterior tangent lines drawn along the vertebral bodies at the superior and inferior limits of the spinal segment being evaluated, providing an estimate of the total sagittal curvature of that region.

RRA Segment	Normal Values	X-ray 1 Values	Versus Normal (X-ray1)	X-ray 2 Values	Versus Normal (X-ray 2)	% Change X-ray 1→2
ARA C2-C7	-42.0º	2.1º	105.00%	-20.4º	51.40%	1071.40%
Translation C2-C7	0.0 mm	19.7 mm	19.7 mm	15.5 mm	15.5 mm	21.30%

## Discussion

**Figure 5:**
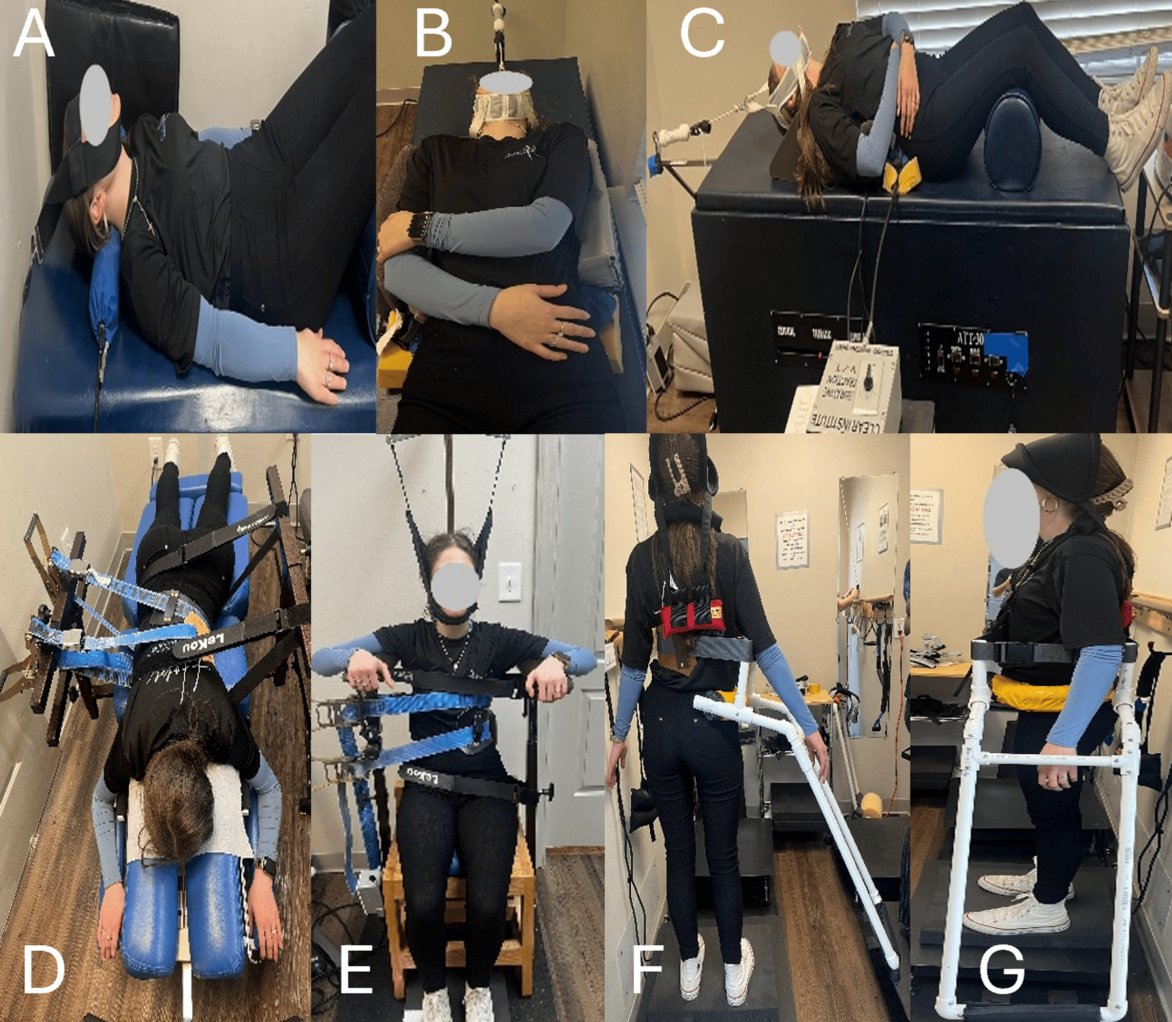
A: Vibrating traction cervical (mechanical traction); B-C: Mechanical drop piece (mechanical traction); D: Flexion/distraction table; E: Scoliosis traction chair; F-G: Spinal weighting (labyrinthine righting reflex) A: Cervical lordosis was improved using mechanical vibrating traction, applying controlled Y-axis distraction. Traction was delivered at 5 pounds with superimposed vibration at 4.5 Hz. This was performed for 20 minutes per session. B-C: A joint-mobilization method designed to restore motion to a restricted or stiff articulating segment vibrating at 4.5Hz, with 10 pounds of Y-axis traction, for 20 minutes. D: A scoliosis-specific therapeutic table applying a low-force, intermittent traction for 20 minutes. E: A seated traction technique designed to provide low-force spinal unloading while progressively promoting spinal de-rotation, elongation, and realignment to reduce scoliotic deformity for 30 minutes. F-G: This approach engages intrinsic postural control pathways by applying external weighting to stimulate compensatory righting responses, including activation of the labyrinthine righting reflex, for 10 minutes. Repeated exposure reinforces these corrective responses, promoting neuromuscular adaptation and long-term postural re-education. Source: [7], reproduced under the CC BY 4.0 deed.

This case series presents two geriatric female patients with long-standing adolescent idiopathic scoliosis who experienced significant clinical improvement following a non-surgical rehabilitation program through the CLEAR Institutes' protocols (Figure 5) [12].

The central finding is not simply that treatment was effective. It is that the most clinically meaningful improvements occurred in the sagittal plane and kinetic chain. This is seen specifically in cervical alignment and forward head posture, rather than in the primary coronal scoliotic curve. The kinetic chain combination of the lumbar extensors, hip extensors, and piriformis activation created much of the imbalance and associated symptoms. This pattern of response supports the hypothesis that the pain in these patients was driven primarily by kinetic chain dysfunction and compensatory degeneration, not by the scoliotic curve itself. This observation is critical, as the relationship between back pain and the magnitude of the scoliotic curve in adults is inconsistent, suggesting other factors are at play [14-17].

Both patients, despite having different Cobb magnitudes, presented with strikingly similar and severe sagittal plane deformities. Case 1 presented with a cervical ARA of 2.1 degrees, a near-complete inversion of the normal cervical lordosis. Case 2 presented with a cervical ARA of -9.2 degrees and a forward head posture of 25.9 mm. These findings are consistent with a whole-body compensatory strategy in which the cervical spine undergoes progressive kyphotic deformation to maintain horizontal gaze in the presence of a primary thoracolumbar or lumbar deformity [18,19]. Prior retrospective cross-sectional analysis of 37 patients with AIS from this clinic demonstrated that all participants exhibited a loss of cervical lordosis [6]. The two cases presented here are consistent with this pattern and extend it to a geriatric population where sagittal malalignment is a known contributor to pain, functional decline, and increased fall risk [19-21].

The kinetic chain concept provides the theoretical framework for understanding why this compensatory cervical deformity is clinically significant. Decades of abnormal cervical kyphosis and forward head posture generate chronic, elevated tensile and compressive loads on the cervical and upper thoracic musculature, facet joints, intervertebral discs, and ligamentous structures [22-26]. These loads accelerate degenerative changes, including facet joint osteoarthritis and fatty infiltration of paraspinal muscles, and create a persistent nociceptive environment [27-31]. The body then adapts the lower trunk and into the lower extremities. Over time, pain may develop as a downstream consequence of chronic biomechanical dysfunction throughout the kinetic chain, not a direct symptom of the scoliotic curve. This distinction has important implications for treatment. A strategy focused solely on reducing the Cobb angle may fail to address the actual source of symptoms [14].

The outcomes observed in these two cases align with findings from the literature on conservative scoliosis care [7,8,11,32]. The CLEAR Institute protocol used in both cases is a structured, multimodal approach to non-surgical scoliosis management that has been shown to produce positive outcomes [1,9-11]. The present cases suggest that in geriatric patients, the protocol's effects on sagittal alignment and the kinetic chain may be particularly relevant to pain outcomes and an increase in the ability to perform activities of daily living. The reduction in forward head posture observed in Case 2, from 25.9 mm to 11.9 mm, is a clinically substantial change that likely reduced the chronic mechanical burden on the cervical spine and is a contributor to the resolution of symptoms [26].

This case series has several important limitations. The sample size of two patients precludes generalization. No control group was included. Functional outcome measures beyond self-reported pain intensity were not systematically collected. The minimal clinically important difference (MCID) of approximately 9 points of the functional rating index was not achieved [33]. The follow-up period was limited to two months. Longer-term follow-up data would be necessary to determine whether the radiographic and clinical improvements observed are sustained. Despite these limitations, the cases provide a coherent and clinically meaningful illustration of the kinetic chain dysfunction hypothesis in geriatric AIS patients. They also demonstrate that non-surgical rehabilitation targeting global spinal alignment may be feasible and potentially effective in this population [32,34-35].

## Conclusions

This case series suggests that in geriatric patients with AIS, chronic pain may be more closely related to kinetic chain dysfunction and compensatory sagittal deformity than to the primary scoliotic curve. These findings underscore the importance of assessing global spinal biomechanics in this population. A non-surgical, multimodal rehabilitation program based on the CLEAR Institute's protocol produced clinically meaningful reductions in pain and measurable improvements in sagittal spinal alignment in both patients. These findings support the hypothesis that treatment in this population should prioritize restoration of global biomechanical function. Larger prospective studies are needed to confirm these observations and to characterize the long-term durability of non-surgical rehabilitation in geriatric patients with AIS.
